# Impact of Nectar Composition and Nectar Yeasts on Volatile Emissions and Parasitoid Behavior

**DOI:** 10.1007/s10886-025-01587-1

**Published:** 2025-03-06

**Authors:** Islam S. Sobhy, Tim Goelen, Felix Wäckers, Kevin J. Verstrepen, Tom Wenseleers, Hans Jacquemyn, Bart Lievens

**Affiliations:** 1 CMPG Laboratory for Process Microbial Ecology and Bioinspirational Management (PME&BIM), Department of Microbial and Molecular Systems, KU Leuven, Leuven, Belgium; 2 Department of Plant Protection, Faculty of Agriculture, Suez Canal University, Ismailia, Egypt; 3 Biobest, Westerlo, Belgium; 4 Lancaster Environment Centre, Lancaster University, Lancaster, UK; 5 VIB – KU Leuvain Center for Microbiology, Leuven, Belgium; 6 CMPG Laboratory of Genetics and Genomics, Department of Microbial and Molecular Systems, KU Leuven, Leuvain, Belgium; 7 Laboratory of Socio-Ecology & Social Evolution, Biology Department, KU Leuven, Leuvain, Belgium; 8 Laboratory of Plant Conservation and Population Biology, Biology Department, KU Leuven, Leuvain, Belgium; 9 KU Leuven Plant Institute (LPI), KU Leuven, Leuvain, Belgium; 10 Present Address: School of Biosciences, Cardiff University, Museum Avenue, Cardiff, UK; 11 Present Address: Flemish Institute for Technological Research (VITO), Mol, Belgium

**Keywords:** *Aphidius ervi*, *Metschnikowia gruessii*, *Metschnikowia reukaufii*, Microbial volatiles, Olfactory response, Pest management

## Abstract

Nectar yeasts can significantly influence the scent of floral nectar and therefore the foraging behavior of flower-visiting insects. While these effects likely depend on nectar chemistry and yeast species, their joint impact on nectar volatile profiles and associated insect responses remain poorly understood. Here, we used four synthetic nectar types varying in sugar and amino acid concentration and two specialist nectar yeasts (*Metschnikowia gruessii* and *Metschnikowia reukaufii*) to investigate how nectar composition and yeast species affect volatile profiles and the olfactory responses of the generalist aphid parasitoid *Aphidius ervi*. Olfactometer assays showed that *A. ervi* females significantly preferred fermented nectars with high amino acid-low sugar content (HL) and low amino acid-high sugar (LH) content, regardless being fermented by *M. gruessii* or *M. reukaufii*, over non-inoculated nectars. This effect was not observed for nectars with low amino acid-low sugar (LL) and high amino acid-high sugar (HH) content*.* Moreover, LL nectar fermented with *M. gruessii* became even repellent to the parasitoids. GC–MS analysis of volatile organic compounds (VOCs) revealed that VOC profiles of fermented nectars depended significantly on nectar type (i.e., chemical composition), yeast species, and their interaction. Whereas propyl acetate, isobutyl acetate, styrene, α-guaiene and pentyl-octanoate were associated with the LH fermented nectars, ethyl acetate and *E*-methyl isoeugenol were mainly associated with the HL fermented nectars, suggesting possible involvement in *A. ervi* attraction to these nectars. In contrast, isopropyl-hexadecanoate was associated with the non-attractive or repellent LL fermented nectars. Altogether, our results indicate that nectar composition has a strong impact on nectar scent when fermented by specialist nectar yeasts and subsequently on insect foraging behavior.

**Supplementary Information:**

The online version contains supplementary material available at 10.1007/s10886-025-01587-1.

## Introduction

Microorganisms are key contributors to the phenotype of plants (Hawkes et al. 2021), and may therefore strongly impact their ecological interactions (Friesen 2011). One group of microorganisms that has gained increased attention as key players in manipulating plant chemical phenotypes is those inhabiting floral nectar (Álvarez-Pérez et al. 2019; Vannette 2020). This group includes various yeast and bacterial species that have developed specific adaptations that enable them to survive and proliferate in the harsh environmental conditions of floral nectar (Lievens et al. 2015). Floral nectar is generally characterized by moderate to high concentrations of sugars (Nicolson 2022), leading to high osmotic pressure and low water activity (Lievens et al. 2015). In addition to sugars, floral nectar also contains smaller amounts of amino acids, lipids and minerals (Nicolson 2022).

Microorganisms colonizing floral nectar can modify various nectar traits, including the concentration and composition of sugars and amino acids (Canto et al. 2017; Nepi et al. 2018), the content of secondary metabolites (Vannette and Fukami 2016), pH (Vannette et al. 2013) and even temperature, leading to less viscous nectar (Herrera and Pozo 2010). Evidence is accumulating that nectar-inhabiting microbes also influence nectar volatile composition (Golonka et al. 2014; Rering et al. 2018, 2020; Sobhy et al. 2018, 2019; Schaeffer et al. 2019; Cusumano et al. 2023; Ermio et al. 2024). These changes in nectar chemistry and volatile composition can have a strong impact on the foraging behavior of flower-visiting insects such as pollinators and parasitoids (Rering et al. 2018; Sobhy et al. 2018; Schaeffer et al. 2019; Martin et al. 2022; Cusumano and Lievens 2023; Cusumano et al. 2023; Ermio et al. 2024), potentially affecting plant reproductive traits such as seed number (Herrera et al. 2013; Schaeffer et al. 2014; Pozo et al. 2015) and enhancing indirect defense against herbivores by promoting biological pest control, respectively (Lenaerts et al. 2016; Álvarez-Pérez et al. 2024; Sobhy et al. 2024).

Nectar yeasts commonly emit a plethora of volatile organic compounds (VOCs), most of which are the result of the metabolism of carbohydrates, amino acids and lipids (Dzialo et al. 2017). As floral nectar varies considerably in sugar and amino acid content and composition across plant species (Venjakob et al. 2021; Nicolson 2022), it is likely that the VOCs produced by yeast fermentation – and the corresponding olfactory responses of flower-visiting insects – differ between plant species. This variation is most likely shaped by both nectar composition and the yeast species colonizing the nectar (Dzialo et al. 2017; Gonzalez et al. 2019). However, so far only very little is known about how nectar chemistry and the metabolic activity of nectar-inhabiting microbes affect the volatile composition of nectar and, in turn, insect behavior.

In this study, we investigated how nectar-inhabiting yeasts affect the VOC profiles of nectars with varying sugar and amino acid contents, and how these differences affect the behavior of flower-visiting insects. Specifically, we examined the effects of two specialist nectar yeasts, *Metschnikowia gruessii* and *Metschnikowia reukaufii*, on the VOC profiles of four synthetic nectars, each differing in sugar and amino acid concentrations. Subsequently, we assessed how these changes influenced the olfactory response of a generalist nectar-feeding parasitoid, *Aphidius ervi*.

## Methods and Materials

### Study Species

#### Yeasts

Experiments were performed with the ascomycetous yeast species *Metschnikowia gruessii* (ST12.14/016) and *M. reukaufii* (ST12.14/017) (Sobhy et al. 2018). Both species belong to the genus *Metschnikowia*, which contains > 80 species, most of which are widely distributed (de Vega et al. 2018). *Metschnikowia gruessii* and *M. reukaufii* are the most commonly found yeasts in floral nectar (Herrera et al. 2009). Their high prevalence in floral nectar has been attributed to their ability to thrive in environments with high C/N ratios and their capacity to efficiently exploit a wide diversity of resources (Pozo et al. 2015). Both species are typically dispersed between flowers by nectar-foraging insects (Brysch-Herzberg 2004) and can reach high densities (10^4^–10^5^ cells µL^−1^) in floral nectar within a few days after inoculation (Herrera et al. 2009). Nectar fermented by these yeast species not only attracts flower-visiting insects (Rering et al. 2018; Sobhy et al. 2018; Yang et al. 2019; Crowley-Gall et al. 2021; Ermio et al. 2024), but the associated odors also serve as effective learning cues for generalist nectar foragers (Sobhy et al. 2019).

#### Insects

Behavioral experiments were performed with female adults of the generalist aphid parasitoid *Aphidius ervi* Haliday (Hymenoptera: Braconidae). The species is widely distributed and commonly used for biological control of aphids in greenhouses (van Lenteren 2012). The immature stages of *A. ervi* develop within aphid hosts, ultimately killing them, while adult parasitoids primarily feed on floral nectar and aphid honeydew to meet their energetic and nutritional needs (Vollhardt et al. 2010). Previous research has shown that adult females of *A. ervi* are significantly attracted to nectars fermented by *M. gruessii* and *M. reukaufii*, with a stronger preference to the latter (Sobhy et al. 2018). In a meta-analysis on parasitoid foraging behavior, Zemenick et al. (2019) reported that parasitoids were present in almost half of the analyzed flower-visitor datasets. Across all datasets, flower-visitor networks included parasitoid species from 14 different families, with Braconidae representing the second-largest group (73 flower-visiting species), supporting the close association between parasitoids and flowers (Zemenick et al. 2019).

### Preparation of Yeast-Fermented Nectars

We prepared four synthetic nectar solutions that mimic the variation in sugar and amino acid concentrations that yeasts may encounter in natural floral nectar (Lievens et al. 2015). These synthetic nectars represented different combinations of low (0.0316 mM) or high concentrations of amino acids (3.16 mM) and low (15%; 0.15 g mL^−1^) or high concentrations of sucrose (50%; 0.5 g mL^−1^) (Vannette and Fukami 2014). This resulted in four different nectar types: low amino acid–low sugar nectar (LL), low amino acid–high sugar nectar (LH), high amino acid–low sugar nectar (HL), and high amino acid–high sugar nectar (HH) (Table S1, Supporting Information). We used digested casein as a source of amino acids, as its composition closely resembles that of nectar amino acids (Vannette and Fukami 2014, 2016). Prior to the fermentations, all nectar solutions were sterilized by filtration through a 0.22 µm filter (Nalgene, Waltham, MA, USA).

Yeast-fermented nectars were prepared following the procedure outlined in Sobhy et al. (2018). Briefly, yeast strains were revived from cryopreservation at −80 °C by plating stock cultures on yeast extract peptone dextrose agar (YPDA), then inoculated in test tubes containing 5 mL yeast peptone dextrose broth (YPDB) and incubated overnight at 25 °C on a rotary shaker at 150 rpm. Cells were then washed and resuspended in sterile saline water (0.9% NaCl) until an optical density (OD 600 nm) of 1 was reached. A 1.5 mL aliquot of the suspension was used to inoculate 150 mL of sterile synthetic nectar in a 250 mL Erlenmeyer flask. The flasks were sealed with fermentation water locks and incubated at 25 °C for seven days under static conditions. This setup created an environment that allowed pressure to escape during fermentation, while preventing the entry of external air, contaminants, and microbes, thereby supporting optimal yeast growth and volatile production (Fleet 1998). A 7-day fermentation period was chosen to obtain yeast densities similar to those in natural floral nectar (> 10^4^ cells μL^−1^) (de Vega et al. 2009; Herrera et al. 2009). For each nectar-yeast combination, three independent fermentations were performed, and an additional nectar solution without yeast inoculation (*n* = 3) was included as a control (sterility of the control medium was confirmed by plating after the incubation period). Following the incubation period, nectars were centrifuged at 10,000 × g for 15 min, filtered through 0.22 µm filter (Nalgene, Waltham, MA, USA) to obtain cell-free cultures, and stored in small aliquots in sterile dark glass vials (Fagron, Nazareth, Belgium) at −20 °C until further use.

### Chemical Analysis of Yeast-Fermented Nectars

VOC profiles of all nectar samples were analyzed using headspace solid-phase microextraction gas chromatography-mass spectrometry (HS–SPME–GC–MS). For each sample, 10 mL of cell-free nectar was placed in a 20 mL glass vial with 1.75 g of NaCl and incubated at 60 °C with continuous agitation using a TriPlus RSH SPME auto sampler (Thermo Fisher Scientific, Waltham, Massachusetts, USA) to promote emission of volatiles from the nectar (Yang et al. 2021). Volatiles were extracted from the headspace with a 50/30 μm DVB/CAR/ PDMS coated SPME fiber (Supelco, Bellefonte, Pennsylvania, USA), which was conditioned with a pre-desorption time of 2 min and a post-desorption time of 5 min at 250 °C. Samples were analyzed on a Thermo Trace 1300 GC system. Fibers were desorbed in the injection port at 270 °C in splitless mode for 3 min (Goelen et al. 2020a) and analyzed with a MXT-5 column (30 m length × 0.18 mm inner diameter × 0.18 μm film thickness; Restek Bellefonte, Pennsylvania, USA). A pulsed helium gas flow was programmed for injection, starting at 2.7 mL min^−1^ for 0.1 min, and then decreasing to 0.9 mL min^−1^. The oven temperature program was as follows: 30 °C for 3 min, then increasing at 7 °C min^−1^ to 80 °C, then at 2 °C min^−1^ to 125 °C, and finally at 8 °C min^−1^ to 270 °C, where it was held for 15 min. Mass spectra were recorded with a single quadrupole mass spectrometer (Thermo Fisher Scientific) in centroid mode using a mass acquisition range of 33–550 atomic mass units, a scan rate of 5 scans per second, and an electron impact ionization energy of 70 eV. A mix of linear n-alkanes (from C7 to C23; Supelco) was injected into the GC–MS as external retention index markers under identical conditions.

Compounds were putatively identified and quantified as described in Ermio et al. (2024). Briefly, chromatograms were processed using AMDIS 32 for peak deconvolution to resolve overlapping signals. The resulting spectra were searched with NIST MS Search v2.0 g software against the NIST2017, FFNSC and Adams libraries. Peak areas were compared to a background signal obtained from a GC–MS analysis of demineralized water. This background signal was subtracted from the peak areas of the corresponding tentatively identified compounds in nectar samples. Compounds with peak area differences below 1,000 and/or that only appeared in one of the three replicates were excluded from further analysis. To refine the extraction and integration of the elution profiles, a custom file containing retention times and spectral profiles of target compounds was used. Extraction was performed for each compound in every chromatogram over a defined time window using weighted non-negative least squares analysis (Lawson and Hanson 1995). For every compound, the peak areas were calculated from the extracted profiles.

### Olfactometer Bioassays

To investigate the extent to which fermented nectars with varying sugar and amino acid composition influenced the olfactory response of parasitoids, naïve *A. ervi* females (< 24 h old and inexperienced to smell and food) were tested using a Y-tube olfactometer bioassay following the protocol described by Sobhy et al. (2018). Parasitoids were supplied as mummies by a commercial biocontrol company (Ervi-system®, Biobest, Westerlo, Belgium). Upon receipt, the mummies were placed in a nylon insect cage (20 × 20 × 20 cm, BugDorm-41515, MegaView Science Co., Ltd, Taichung, Taiwan) and maintained under controlled conditions (22 °C, 70% relative humidity, 16:8 h photoperiod) until adult emergence. In the experiments, female parasitoids were given a choice between the odor of fermented nectar and the corresponding unfermented nectar (control).

The Y-tube olfactometer (stem: 20 cm; arms: 12 cm with a 60° angle at the Y-junction; inner diameter: 1.5 cm) was positioned on a table that was homogeneously illuminated by four 24W T5 TL-fluorescent tubes (16 × 549 mm, 1350 Lumen, 5500 K, True-Light®, Naturalite Benelux) with a 96% colour representation of true daylight at a height of 45 cm. The Y-tube was mounted at a 20° incline to stimulate parasitoid movement towards the Y-junction. Charcoal-filtered, humidified air was provided at a rate of 400 mL min^−1^ (Brooks Instrument flow meter, Hatfield, USA) to both arms of the Y-tube using a vacuum pump (Tetratec APS 150, Mella, Germany). To eliminate any visual cues that might influence insect response, the olfactometer was completely enclosed with white curtains.

To assess the parasitoid's preference for one of the two nectar options, 150 µL of cell-free fermented nectar was loaded onto a filter paper (Ø 37 mm, Macherey–Nagel, Düren, Germany) and placed in one of the olfactometer odor chambers, while another filter paper with 150 µL of control nectar was placed in the second chamber. To evaluate whether unfermented nectar affected parasitoid behavior, the same assay was performed where the parasitoids were given the choice between unfermented nectar and sterile distilled water.

For each nectar-yeast combination, the bioassay was carried out by releasing 200 adult females in 40 groups of five individuals (20 groups per day). Parasitoids were released at the base of the olfactometer, and their responses were recorded ten minutes later. Wasps that crossed a set line in of one of the olfactometer arms (1 cm from the Y-junction) at the time of evaluation (10 min following the release) were recorded as having chosen the odor source presented in that olfactometer arm (Sobhy et al. 2018). Parasitoids that did not make a choice within 10 min after release were considered as non-responders and excluded from the statistical analysis. Each parasitoid was tested only once.

To avoid positional bias, the odor chambers were rotated after every ten releases, and a new set of Teflon tubes was used. Simultaneously, the Y-tube was replaced with a cleaned one. Filter papers with 150 μL of the tested medium were replaced with fresh ones every two runs to ensure consistent odor release. At the end of each experiment, all olfactometer parts were thoroughly cleaned with tap water, distilled water, acetone (Forever, Courcelles, Belgium; purity > 99%), and finally pentane (Sigma-Aldrich, Steinheim, Germany; purity 98%). After solvents had evaporated, the glass parts were placed overnight in an oven at 150 °C. All bioassays were conducted over two consecutive days for each tested combination. Preliminary experiments confirmed that conducting the bioassays on separate days did not influence parasitoid behavior, as all tests were carried out under highly controlled conditions of 22 °C and 70% RH between 09:00 h and 16:00 h.

### Data Analysis

To obtain an overview of the variation in volatile profiles, a Principal Component Analysis (PCA) was performed using the absolute peak areas of the detected volatiles as dependent variables in MetaboAnalyst 6.0 (Pang et al. 2024). Prior to analysis, VOC data were log-transformed and auto-scaled (i.e. mean-centered and divided by the standard deviation of each variable). To assess the effect of nectar type and yeast fermentation on volatile profiles, a permutational multivariate analysis of variance (PERMANOVA) was performed on the transformed data with nectar type, yeast species and their interaction as fixed factors using the adonis2 function in the R package vegan (Oksanen et al. 2023). Additionally, one-way ANOVAs were applied to the key volatile compounds differentiating the various nectars as identified in the PCA. For the univariate analysis, normality of the data was first tested using the Shapiro–Wilk test and homogeneity of variances through Levene's test. If these assumptions were not met, a non-parametric Kruskal–Wallis ANOVA on ranks was used. Post-hoc comparisons among means were performed using Tukey. *P*-values were adjusted for multiple comparisons using the Bonferroni correction method to control the false discovery rate (FDR) and ensure robust statistical inference. Both univariate and multivariate analyses were performed using SigmaPlot version 15.0 (Systat Software, San Jose, CA, USA).

For each olfactometer bioassay, parasitoid olfactory response was analyzed using a Generalized Linear Mixed Model (GLMM) based on a binomial distribution and a logit link function (logistic regression) using the ‘glmer’ function from the lme4 package in R (Bates et al. 2015). Nectar type, yeast species, and their interaction were used as fixed factors. Each release of five individuals was considered as a replicate. To adjust for overdispersion and to prevent pseudo-replication, each group release (*n* = 40) was included in the model as a random factor. The number of parasitoids choosing the control or treatment side in each cohort was entered as response variable (Goelen et al. 2021). To evaluate the preference of *A. ervi* for the different fermented nectars, we tested the null hypothesis (H_0_) that the parasitoids showed no preference for any olfactometer arm (i.e., 50:50 response) by testing H_0_: logit = 0 which equals a 50:50 distribution. In addition, a Type III Wald chi-square test was performed on the GLMM to determine whether there were overall differences in olfactory responses to the various nectar types fermented by the two yeast species. A significance level of α = 0.05 was used to determine significant attraction or repellence. The resulting *p*-values were corrected for multiple pairwise comparisons using the false discovery rate (FDR) method implemented in the R function “*p*.adjust”. The analysis of parasitoid olfactory responses was performed in R (R Core Development Team 2021).

## Results

### Nectar Volatile Profiles

GC–MS analysis detected 36 tentatively identified volatile compounds from four main chemical classes (i.e., alcohols, benzenoids, esters, and terpenoids), amongst some others, in the headspace collections of the different nectars (Table S2, Supporting Information). Principal component analysis (PCA) of the volatile data showed that the first two principal components (PC1 and PC2) accounted for 74.54% of the total variation in the data. A clear separation was observed along PC1 between the volatile profiles of control nectars and those fermented by the yeasts. PC2 further separated LH nectar fermented by *M. reukaufii*, LH nectar fermented by *M. gruessii* and, to a lesser extent, HH nectar fermented by *M. reukaufii* from the other fermented nectars (Figure S2, Supporting Information). The PERMANOVA analysis indicated that nectar type (*F*_*3,24*_ = 13.577; *R*^*2*^ = 0.09014; *P* < 0.001), yeast species (*F*_*2,24*_ = 162.926; *R*^*2*^ = 0.72116; *P* < 0.001) and their interaction (*F*_*6,24*_ = 10.211; *R*^*2*^ = 0.13559; *P* < 0.001) had a significant effect on the VOC composition of the different nectars.

**Fig. 1:**
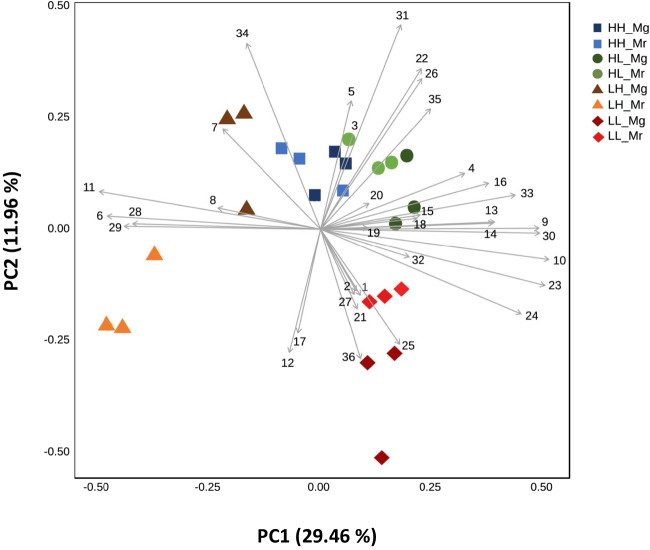
Principal Component Analysis (PCA) of the volatile profiles emitted from different synthetic nectars (*i.e.*: HH, high amino acid and high sugar content; HL, high amino acid and low sugar content; LH, low amino acid and high sugar content; LL, low amino acid and low sugar content), which were fermented by either *Metschnikowia gruessii* (Mg) or *M. reukaufii* (Mr). The Biplot visualizes the ordination of the different samples according to the first two principal components (PCs) based on the quantity of the volatiles emitted from the nectars. Vectors (in grey) visualize the loadings for each variable. Vector numbers refer to the different volatile compounds: (1) isopropylalcohol, (2) ethanol, (3) 2-butanone, (4) ethyl acetate, (5) isobutanol, (6) propyl acetate, (7) ethyl isobutyrate, (8) isobutyl acetate, (9) methylpyrazine, (10) 2,3-butanediol, (11) styrene, (12) 3-methyl-2-hexanol, (13) amyl acetate, (14) ethyl tiglate, (15) benzaldehyde, (16) 5-methyl-2-furanmethanol, (17) 1-octen-3-ol, (18) isopentyl butanoate, (19) 2-ethyl-1-hexanol, (20) benzyl alcohol, (21) (*E*)-β-ocimene, (22) prenyl isobutyrate, (23) undecane, (24) phenethyl alcohol, (25) 4-methyl-1-pentanol, (26) isoborneol, (27) 1-α-terpineol, (28) α-guaiene, (29) pentyl-octanoate, (30) 9-epi-(*E*)-caryophyllene, (31) ethyl-(*E*)-cinnamate, (32) trans-calamenene, (33) *E*-methyl isoeugenol, (34) ethyl dodecanoate, (35) 14-hydroxy-α-humulene, and (36) isopropyl-hexadecanoate. All analyses were performed on cell-free nectar solutions (three biological replicates; *n* = 3). Volatile data were log-transformed and auto-scaled (mean-centered and divided by the standard deviation of each variable) prior to analysis

Since the non-fermented nectar emitted minimal volatiles (Table S2, Supporting Information), an additional PCA was conducted exclusively on the fermented nectars to identify VOCs associated with and/or differentiating the various nectars. The PCA revealed distinct clustering along PC1 (explaining 29.46% of the variation), particularly separating the volatile profiles of LH nectar fermented by *M. reukaufii* and *M. gruessii*, with a clear distinction between both, the fermented HH nectars, and the other fermented nectars. A less pronounced separation was observed along PC1 between the fermented HL and LL nectars (Fig. 1). PC2 (explaining 11.96% of the variation) effectively distinguished LL nectar fermented by *M. reukaufii* and *M. gruessii*, along with LH nectar fermented by *M. reukaufii*, from the remaining nectar samples (Fig. 1). PERMANOVA analysis revealed that nectar type (*F*_*3,16*_ = 12.8160; *R*^*2*^ = 0.41494; *P* < 0.001), yeast species (*F*_*1,16*_ = 8.6539; *R*^*2*^ = 0.09339; *P* < 0.001) and their interaction (*F*_*3,16*_ = 9.8527; *R*^*2*^ = 0.31899; *P* < 0.001) had a significant effect on the volatile profiles emitted by the different fermented nectars.

The biplot further shows that propyl acetate (6), isobutyl acetate (8), styrene (11), α-guaiene (28) and pentyl-octanoate (29) were strongly associated with fermented LH nectars, whereas ethyl acetate (4), 5-methyl-2-furanmethanol (16), benzyl alcohol (20), *E*-methyl isoeugenol (33) and 14-hydroxy-α-humulene (35) were mainly linked to fermented HL nectars. In contrast, (*E*)-β-ocimene (21), 4-methyl-1-pentanol (25), α-terpineol (27) and isopropyl-hexadecanoate (36) were primarily associated with fermented LL nectars (Fig. 1). Supporting these findings, propyl acetate (*F*_*7,16*_ = 27.635, *P* < 0.001), isobutyl acetate (*H*_*7*_ = 21.023, *P* = 0.004), styrene (*F*_*7,16*_ = 19.434, *P* < 0.001), α-guaiene (*F*_7, 16_ = 4.756, *P* = 0.005), and pentyl-octanoate (*F*_7, 16_ = 12.803, *P* < 0.001) were emitted at significantly higher levels from fermented LH nectars. Similarly, ethyl acetate (*H*_*7*_ = 16.160, *P* = 0.024) and *E*-methyl isoeugenol (*F*_7, 16_ = 7.521, *P* < 0.001) were emitted in significantly higher amounts from fermented HL nectars, while isopropyl-hexadecanoate (*H*_*7*_ = 17.086, *P* = 0.017) was emitted in significantly higher amounts from fermented LL nectars (Table 1). However, despite these general patterns notable differences were observed in the emission of specific volatile compounds depending on the yeast species. For instance, LH nectar fermented by *M. reukaufii* exhibited significantly higher emissions of isobutyl acetate, propyl acetate, and pentyl octanoate, by approximately sevenfold, sixfold, and twofold, respectively, in comparison with LH nectar fermented by *M. gruessii*. In contrast, HL nectar fermented by *M. gruessii* produced significantly higher levels of isobutyl acetate (threefold) compared to HL nectar inoculated with *M. reukaufii*. Likewise, LL nectar fermented by *M. gruessii* emitted higher amounts of the monoterpenoids (*E*)-β-ocimene (fourfold) and α-terpineol (1.3-fold) (Table 1).

**Table 1:** Volatile organic compound (VOC) composition^*^ of the cell-free nectars fermented by the nectar yeasts used in this study

Volatiles	RI	HH		HL		LH		LL	
		Mg	Mr	Mg	Mr	Mg	Mr	Mg	Mr
Alcohols									
ethanol	445	140 ± 47.8	162 ± 6.3	257 ± 19.3	253 ± 8.3	206 ± 2.2	227 ± 12.4	255 ± 10.9	270 ± 10.7
isopropyl alcohol	536	575.6 ± 33.1	516.5 ± 8.3	1097 ± 79.8	1108 ± 33.3	944 ± 54.4	946.0 ± 30.4	924 ± 22.3	1088.2 ± 23.1
isobutanol	653	70.1 ± 20.8	18.7 ± 7.7	60.1 ± 7.5	52.9 ± 36	120 ± 6.0	27.3 ± 22.3	9.9 ± 8.0	109 ± 9.5
2,3-butanediol	753	57.3 ± 14.8	44.5 ± 2.2	81.9 ± 25.6	110 ± 3.8	9.6 ± 7.8	ND	79.6 ± 5.9	103 ± 6.5
3-methyl-2-hexanol	832	46.2 ± 3.1	41.9 ± 2.2	32.3 ± 7.3	42.7 ± 3.8	47.2 ± 13.9	50 ± 4.8	46.9 ± 1.3	53 ± 4.1
**4-methyl-1-pentanol**	**868**	**6732 ± 1531**	**8966 ± 1567**	**9488 ± 485**	**11,355 ± 3662**	**7261 ± 155**	**7211 ± 515**	**14,681 ± 4600**	**12,916 ± 2578**
**5-methyl-2-furanmethanol**	**975**	**3019 ± 557**^**a**^	**2070 ± 337**^**a**^	**2186 ± 802**^**a**^	**3131 ± 163**^**a**^	**1917 ± 185**^**a**^	**ND**^**b**^	**4018 ± 636**^**a**^	**2248 ± 1142**^**a**^
1-octen-3-ol	981	248 ± 22.6	243 ± 278	340 ± 17.8	357 ± 10.3	272 ± 17.8	5056 ± 390	505.4 ± 13.3	9255 ± 7157
2-ethyl-1-hexanol	1031	12,616 ± 1643	4329 ± 2105	12,268 ± 752	16,056 ± 154	ND	7688 ± 736	7189 ± 8.30	ND
**benzyl alcohol**	**1036**	**62.8 ± 26.2**	**96.5 ± 9.0**	**1648 ± 1237**	**169 ± 1.8**	**676 ± 470**	**108 ± 6.8**	**2086 ± 1636**	**199 ± 0.4**
phenylethyl alcohol	1114	138 ± 33.7	118 ± 13.5	241 ± 38.5	1975 ± 446	23.4 ± 9.5	51.2 ± 9.8	745 ± 14.4	789 ± 3.8
Isoborneol	1162	2284 ± 116.7	17,037 ± 9095	4347 ± 1731	36,355 ± 4883	5406 ± 473	ND	ND	7555 ± 1702
Benzenoids									
**styrene**	**891**	**188 ± 12.4**^**b**^	**173 ± 12.8**^**b**^	**136 ± 13.5**^**c**^	**128 ± 5.1**^**c**^	**567 ± 148**^**a**^	**613 ± 35.2**^**a**^	**118 ± 4.3**^**c**^	**138 ± 4.1**^**c**^
***E*-methyl** **isoeugenol**	**1499**	**2271 ± 44.2**^**ab**^	**2646 ± 320**^**ab**^	**3084 ± 144**^**a**^	**3788 ± 14.6**^**a**^	**2922 ± 56.7**^**ab**^	**1369 ± 377**^**b**^	**3395 ± 78.2**^**a**^	**3572 ± 140**^**a**^
Esters									
**ethyl acetate**	**613**	**100 ± 4.2**^**b**^	**115 ± 8.8**^**b**^	**172 ± 5.7**^**ab**^	**167 ± 16.3**^**ab**^	**60.4 ± 2.0**^**c**^	**34.6 ± 11**^**c**^	**130 ± 54.6**^**b**^	**217 ± 12.5**^**a**^
**propyl acetate**	**712**	**194 ± 2.4 **^**b**^	**193 ± 17.6**^**b**^	**78 ± 1.5**^**c**^	**78.8 ± 4.4**^**c**^	**159 ± 60.3**^**bc**^	**875 ± 60.6**^**a**^	**64.8 ± 6.4**^**c**^	**66.3 ± 1.5**^**c**^
ethyl isobutyrate	755	69.2 ± 5.2	281 ± 19.9	118 ± 34.6	194 ± 6.9	627 ± 194	241 ± 14.6	139 ± 57.1	129 ± 31.7
**isobutyl acetate**	**780**	**ND**^**d**^	**297 ± 48.3**^**a**^	**212 ± 4.4**^**b**^	**71.3 ± 21.9**^**c**^	**46.7 ± 5.2**^**c**^	**310 ± 18.6**^**a**^	**46.9 ± 5.8**^**c**^	**ND**^**d**^
amyl acetate	916	30.5 ± 1.9	27.4 ± 6.1	59 ± 5.3	61.4 ± 4.4	33.3 ± 2.5	19.8 ± 8.1	65.4 ± 15.7	64.1 ± 6.6
ethyl tiglate	926	151 ± 13.1	247 ± 156	317 ± 53.5	293 ± 47.1	40.8 ± 16.8	68.6 ± 1.8	160 ± 14.2	215 ± 10.8
isopentyl butanoate	1041	13,582 ± 2890	7831 ± 858	1238 ± 654	9375 ± 767	7353 ± 610	6258 ± 5093	10,473 ± 4070	9979 ± 411
prenyl isobutyrate	1053	43.5 ± 8.5	212 ± 21.2	344 ± 216	86.5 ± 16.1	40.5 ± 33.1	ND	ND	27.1 ± 11.9
ethyl-(*E*)-cinnamate	1443	206 ± 9.3	127 ± 4.4	310 ± 22.1	271 ± 23.0	71.8 ± 11.9	ND	ND	ND
**pentyl octanoate**	**1468**	**182 ± 10.5**^**b**^	**188 ± 20.4**^**b**^	**179 ± 12.7**^**b**^	**195 ± 6.7**^**b**^	**209 ± 18.0**^**b**^	**501 ± 36.6**^**a**^	**118 ± 22.9**^**c**^	**166 ± 12.9**^**bc**^
ethyl dodecanoate	1582	796 ± 68.9	934 ± 147	676 ± 42.7	760 ± 25.9	1052 ± 192	932 ± 289	ND	708 ± 20.0
**isopropyl-hexadecanoate**	**1827**	**86.6 ± 12.3**^**b**^	**110 ± 38.0**^**ab**^	**ND**^**c**^	**140 ± 17.7**^**ab**^	**ND**^**c**^	**62.7 ± 10.5**^**b**^	**194 ± 95.4**^**a**^	**152 ± 35.1**^**ab**^
Terpenoids									
** (*E*)-β-ocimene**	**1051**	**63.5 ± 6.2**^**b**^	**ND**^**c**^	**861 ± 615**^**a**^	**125 ± 35.6**^**ab**^	**98.7 ± 41.2**^**ab**^	**211 ± 66.2**^**ab**^	**549 ± 367**^**a**^	**126 ± 11.0**^**ab**^
** α-terpineol**	**1143**	**270 ± 24.4**	**273 ± 22.7**	**561 ± 216**	**582 ± 27.4**	**414 ± 44.3**	**378 ± 9.7**	**725 ± 19.5**	**538 ± 195**
** α-guaiene**	**1436**	**84.4 ± 17.4**^**c**^	**132 ± 31.5**^**b**^	**70.9 ± 3.2**^**c**^	**86.4 ± 5.3**^**c**^	**159 ± 37.9**^**ab**^	**256 ± 17.0**^**a**^	**73.1 ± 8.4**^**c**^	**97.8 ± 2.4**^**c**^
trans-calamenene	1508	3223 ± 32.1	3020 ± 1067	ND	5401 ± 39.1	282 ± 63.7	137 ± 35.1	1184 ± 211	2692 ± 1021
9-epi-(*E*)-caryophyllene	1677	273 ± 56.1	362 ± 48.5	590 ± 54.1	710 ± 60.2	381 ± 10.5	31.7 ± 12.9	1090 ± 257	1273 ± 56.1
**14-hydroxy-α-humulene**	**1724**	**339 ± 106**^**ab**^	**ND**^**c**^	**363 ± 40.6**^**ab**^	**469 ± 40.8**^**a**^	**360 ± 46.6**^**ab**^	**ND**^**c**^	**290 ± 120**^**b**^	**529 ± 92.6**^**a**^
Miscellaneous									
2-butanone	597	649 ± 53.1	1042 ± 108	2387 ± 53.8	2781 ± 91.4	534 ± 42.2	646 ± 11.6	1972 ± 814	ND
methylpyrazine	831	73.0 ± 3.9	45.1 ± 18.4	97.9 ± 9.6	86.8 ± 5.3	ND	ND	62.2 ± 0.4	58.6 ± 1.4
Benzaldehyde	953	277 ± 112	663 ± 295	267 ± 89.3	289 ± 33.9	98.8 ± 7.2	123 ± 14.5	246 ± 34.1	172 ± 70.6
undecane	1100	54.7 ± 7.1	37.1 ± 15.9	79.5 ± 7.1	74.3 ± 0.3	ND	ND	97.4 ± 8.6	116 ± 3.9
Total		49,264 ± 2801	52,715 ± 13,174	44,335 ± 2868	97,249 ± 7685	32,578 ± 1144	34,503 ± 4699	51,436 ± 6982	55,712 ± 8978

^*^Peak areas and Kovats retention indices (RI) were obtained using a MXT-5 equipped GC–MS. Presented values are means of peak areas (× 10^7^) ± SE of three biological replicates (*n* = 3) of different nectars (*HH* high amino acid and high sugar content; *HL* high amino acid and low sugar content; *LH* low amino acid and high sugar content; *LL* low amino acid and low sugar content). These nectars were either fermented by two specialist nectar yeasts—*Metschnikowia gruessii* (Mg) and *Metschnikowia reukaufii* (Mr)*.* Under each chemical class, VOCs are ordered in accordance with their increasing retention time in the gas chromatograph and retention index. VOCs were tentatively identified using their spectra, Kovats retention indices and matches from the NIST2017, FFNSC, and Adams libraries. Statistical denotes for the VOCs in bold represent the significant differences (One-Way ANOVA) of the key volatile compounds differentiating the various nectars in PC1 and PC2 of the principal component analysis (PCA). (**see **Figs. 1). Bolded VOCs without denotes indicate no significant statistical difference. Volatile data were log-transformed prior to analysis. ND, not detected

### Parasitoid Olfactory Response

**Fig. 2:**
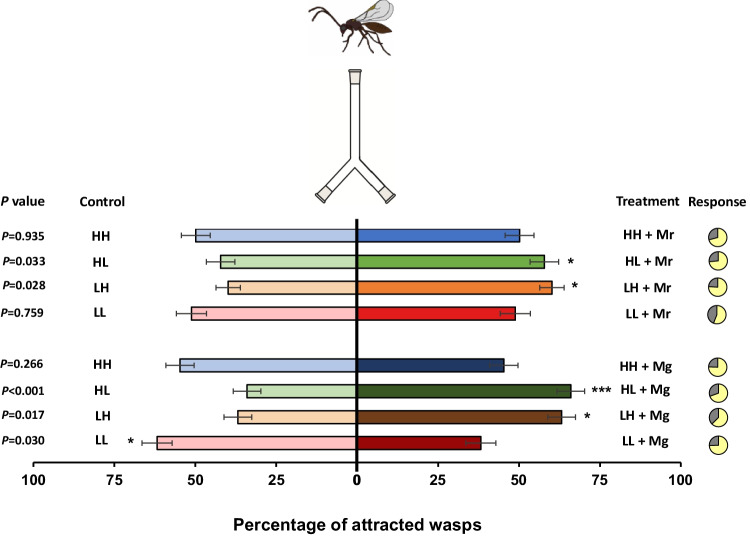
Olfactory response of *Aphidius ervi* females when given the choice between control nectar and fermented nectar (percentage ± SE, *n* = 40). Treatments included four synthetic nectars (*i.e.*: HH, high amino acid and high sugar content; HL, high amino acid and low sugar content; LH, low amino acid and high sugar content; and LL, low amino acid and low sugar content), which were fermented by either *Metschnikowia reukaufii* (Mr) or *M. gruessii* (Mg). Experiments were performed with cell-free nectars. The bioassay was carried out by releasing 40 groups of five females at the base of a two-choice Y-tube olfactometer and evaluating their response ten minutes after their release. The parasitoids used were naïve (*i.e.* inexperienced to odors and honey solutions). Pie charts show the distribution of responding (in yellow) and non-responding (in grey) individuals. Non-responders were eliminated from statistical analysis. Asterisks indicate a preference that is significantly different from a 50:50 distribution within a choice test: *** *P* < 0.001; * 0.01 ≤ *P* ≤ 0.05

Overall, parasitoid responsiveness to the different nectars was higher than 55% (Fig. 2). Parasitoid response was significantly affected by nectar type (*χ*^*2*^ = 16.842; *df* = 3; *P* < 0.001), but not by yeast species (*χ*^*2*^ = 0.086; *df* = 1; *P* = 0.769). The interaction between nectar type and yeast species was also not statistically significant (*χ*^*2*^ = 4.135; *df* = 2; *P* = 0.127). *Aphidius ervi* females were most attracted to fermented HL (*M. gruessii: P* < 0.001; *M. reukaufii*: *P* = 0.033) and LH nectar (*M. gruessii: P* = 0.017; *M. reukaufii: P* = 0.028) (Fig. 2). In contrast, LL nectar fermented by *M. gruessii* elicited a significant negative response, with parasitoid females being more attracted towards the control (*P* = 0.030), while this was not the case for *M. reukaufii* (neutral response; *P* = 0.759). No significant effects were found for fermented HH nectars, irrespective of the yeast species (*M. gruessii*: *P* = 0.266; *M. reukaufii: P* = 0.935). Parasitoid females showed no preference for water or control unfermented nectars (Figure S1, Supporting Information).

## Discussion

In this study, we showed that yeast fermentation affected the VOC profiles of nectar, with the impact of the fermentation being largely dependent on the nectar's sugar and amino acids composition. Differences in VOC profiles, in turn, influenced the olfactory behavior of the aphid parasitoid *A. ervi*, demonstrating a clear link between nectar composition, yeast activity and insect behavior. In the behavioral assays, the parasitoid showed significant attraction to fermented nectars with high amino acid-low sugar content (HL) and fermented nectars with low amino acid-high sugar content (LH), regardless of being fermented by *M. gruessii* or *M. reukaufii.* Moreover, female parasitoids showed a negative response to low amino acid-low sugar content (LL) nectar fermented by *M. gruessii*.

### Yeast Activity and Nectar Composition Affect VOC Profiles

Analysis of the volatile profiles from both yeast-fermented and non-fermented nectars revealed a total of 36 VOCs. Nectars inoculated with *M. gruessii* or *M. reukaufii* emitted significantly higher amounts of volatiles compared to non-inoculated nectar, confirming previous results (Golonka et al. 2014; Rering et al. 2018, 2020; Sobhy et al. 2018, 2019; Yang et al. 2019; Crowley-Gall et al. 2021; Ermio et al. 2024). The identified compounds belonged to four main chemical classes that have been commonly found in floral nectar: alcohols, benzenoids, esters and terpenoids, among some others (e.g. aromatics, ketones, and aldehydes) (Raguso 2004; Kantsa et al. 2018, 2019; Crowley-Gall et al. 2021). Compounds from these classes have also been frequently detected in the volatilome of yeasts (Dzialo et al. 2017) and in yeast-inoculated synthetic nectars (Rering et al. 2018, 2020; Sobhy et al. 2018; Crowley-Gall et al. 2021; Ermio et al. 2024).

Our results also showed that yeast fermentation influenced nectar volatile profiles differently depending on the sugar and amino acid content and their ratio in the nectars. This was especially clear for fermented LH and LL nectars, which were grouped separately from the other fermented nectars in the PCA. It remains unclear why the volatile profiles of the different nectar types were differently affected by yeast fermentation. It is well known that volatile production by yeasts results from the metabolism of sugars and amino acids (Dzialo et al. 2017; Fenner et al. 2022), but further research is needed to determine how different amounts and ratios of these precursors affect volatile profiles. The balance of sugar and amino acids likely affects not only yeast metabolism, but also the types of volatiles they emit. In HL nectars, the high amino acid concentration might push yeast metabolism more toward amino acid-derived volatiles or nitrogenous compounds, whereas in LH nectars yeast fermentation is likely focused more on sugar metabolism, leading to the increased production of alcohols and esters (Dzialo et al. 2017).

Our results also showed differences in the emissions of certain volatiles between *M. reukaufii-* and *M. gruessii*-inoculated nectars. For example, whereas propyl acetate, isobutyl acetate, pentyl octanoate and (*E*)-β-ocimene were emitted in significantly higher amounts from LH nectar fermented by *M. reukaufii*, isobutyl acetate was emitted in significantly higher amounts from HL nectar fermented by *M. gruessii*. This suggests that phylogenetically related yeast species can produce subtly distinct VOC profiles. These observations are in line with previous research showing that *M. gruessii* and *M. reukaufii* display significantly different physiological profiles, including carbon and nitrogen utilization (Pozo et al. 2016).

### Parasitoid Olfactory Response to Various Fermented Nectars

Among the four nectar types tested, female *A. ervi* displayed a significant preference for yeast-fermented LH and HL nectars over non-fermented control nectars, suggesting that these nectars are more attractive. Furthermore, among these fermented nectars, HL nectar fermented by *M. gruessii* was the most attractive nectar. In contrast, parasitoids were not attracted to fermented HH or LL nectars. Moreover, when LL nectar was fermented with *M. gruessii*, it even became repellent to the parasitoid females. Previous research has shown that nectar specialist yeasts such as *M. gruessii* and *M. reukaufii* produce VOC blends that are attractive to bees and parasitoids (Schaeffer et al. 2014; Rering et al. 2018; Sobhy et al. 2018, 2019; Yang et al. 2019; Ermio et al. 2024). However, these studies did not consider the impact of nectar sugar and amino acid content. Our results clearly show that the sugar and amino acids content and ratio in nectar have a strong impact on the olfactory behavior of *A. ervi* when fermented by *M. gruessii* or *M. reukaufii*.

The PCA identified propyl acetate, isobutyl acetate, styrene, α-guaiene and pentyl-octanoate as the major VOCs associated with fermented LH nectar, distinguishing this nectar type from the others. These VOCs have been shown to attract various insect species, including hymenopteran parasitoids. Propyl acetate, for instance, is a common ester emitted by a wide range of yeast species (Ljunggren et al. 2019), and is highly attractive to several insects, including nitidulid beetles (Zilkowski et al. 1999) and *Drosophila* flies (Kleiber et al. 2014), as well as their pupal parasitoid *Trichopria drosophilae* (Đurović et al. 2021). Similarly, isobutyl acetate, an ester emitted from various yeast fermentations, has been shown to attract various insect species such as *Drosophila suzukii* (Scheidler et al. 2015; Cloonan et al. 2019) and nitidulid beetles (Baig et al. 2025). In addition, α-guaiene, a sesquiterpene previously identified in nectar colonized by both *M. reukaufii* and *M. gruessii* (Ermio et al. 2024), has been shown to act as an attractant for a wide range of insects across diverse taxa, including fungus-feeding beetles (Drilling and Dettner 2009), lepidopteran stem borers (Meng et al. 2021; Mo et al. 2023; He et al. 2025), and hymenopteran egg parasitoids (He et al. 2025). Interestingly, Goelen et al. (2021) showed that the related parasitoid wasp *Aphidius colemani* was strongly attracted to styrene in laboratory assays, while van Neerbos et al. (2023) demonstrated that a mixture of styrene and benzaldehyde attracted *A. colemani* over a distance of up to 5 m when applied via dispensers in greenhouses, illustrating its potential to attract aphid parasitoids.

The high attractiveness of fermented HL nectar to *A. ervi* parasitoids may be attributed to the increased emission of ethyl acetate and *E*-methyl isoeugenol which were produced in significantly higher amounts in fermented HL nectar. Previous studies have demonstrated that ethyl acetate not only attracts several insect taxa (e.g. fruit flies, butterflies, sap beetles and stink bugs) but also elicits their electrophysiological responses (Nout and Bartelt 1998; Tang et al. 2013; Christiaens et al. 2014; Akotsen-Mensah et al. 2021). Likewise, methyl isoeugenol has been reported as a common floral VOC from numerous plant orders and is known to attract Tephritid fruit flies (Tan and Nishida 2012; Royer et al. 2018).

Altogether, these results support the idea that these VOCs may attract *A. ervi* to fermented LH and HL nectars, although further research is needed to confirm this. Additional investigations are also required to understand why these compounds were present in higher amounts in fermented LH and HL nectars. Styrene, for example, is commonly produced by the microbial breakdown of phenylalanine (Kim et al. 2019). Therefore, it can be assumed that in LH nectar, where amino acids are limited, phenylalanine is used more efficiently by the yeasts, leading to increased release of styrene. In contrast, in high amino acid environments (HL, HH), phenylalanine may not be as heavily utilized in the same way, leading to lower styrene production. Similarly, compounds such as propyl acetate and isobutyl acetate may be produced more efficiently in LH nectar through prioritized carbohydrate metabolism.

Additionally, it remains unclear why HH and LL nectars did not attract parasitoids and why LL nectar fermented by *M. gruessii* was even repellent. The observed repellent effect could potentially be explained by the high emission of the monoterpenoids (*E*)-β-ocimene and α-terpineol. Previous studies have demonstrated that parasitoids either show no response or are sometimes repelled by volatile blends rich in terpenoids (Mumm and Hilker 2005; Sobhy et al. 2014). Supporting this, innate parasitoid attraction tends to be stronger toward blends with low terpenoid emissions (D’Alessandro et al. 2009). Specifically, (*E*)-β-ocimene has been reported to repel *Spodoptera litura* larvae (Huang et al. 2022), while α-terpineol has demonstrated strong repellency against various insect taxa (Liu et al. 2013; Hieu et al. 2014). Both the ratio and concentration of emitted VOCs play a crucial role in insect behavior, as attraction is not solely dependent on the quantity of VOCs, but also on their quality (i.e. composition and ratio) (Bruce et al. 2010). For instance, low concentrations of certain VOCs, such as terpenoids and aromatics, can enhance parasitoid attraction, while higher concentrations may disrupt or mask important signals (D’Alessandro et al. 2009; Sobhy et al. 2012). While much of the preceding research on volatile masking has focused on plant volatiles (Schröder and Hilker 2008), increasing attention is being directed toward how microbial volatiles can also mask key attractants, whether from plants (Azeem et al. 2015) or other microbes (Verschut et al. 2019). Therefore, understanding these concentration/ratio thresholds is key to interpreting parasitoid behavior towards microbe-fermented nectars (Goelen et al. 2020b).

### Limitations

Although our study clearly provides new insights into the interactions between nectar, nectar-dwelling yeasts, and insects, certain limitations should be considered. First, although our study focused on nectar types differing in sugar (sucrose) and amino acid content, natural nectars typically contain a variety of sugars and amino acids in varying proportions, in addition to trace lipids, inorganic compounds, vitamins, and plant secondary metabolites (Stevenson et al. 2017; Nicolson 2022). These components can influence yeast metabolism, fermentation, and the resulting nectar scent profile (Jacquemyn et al. 2021). Thus, future research should investigate how real nectars affect nectar-inhabiting microorganisms, and in turn insect behavior. Second, while our study focused on two of the most prevalent nectar-inhabiting yeast species, floral nectar can be colonized by a variety of yeast and bacterial species (Lievens et al. 2015; Pozo et al. 2015), which may differ significantly in their effects on volatile profiles (Lenaerts et al. 2017; Sobhy et al. 2018; Cusumano et al. 2023). Third, we assessed effects using monocultures, whereas in nature, nectar is typically inhabited by several interacting species (Álvarez-Pérez et al. 2019). It remains unclear how species-specific effects or microbe-microbe interactions impact insect behavior (Crowley-Gall et al. 2021). One final technical point is our analytical approach, which utilized forced volatile extraction combined with sensitive detection using SPME at 60 °C. This method enabled the identification of 36 volatile compounds. However, it is important to recognize that these profiles may not fully reflect the naturally emitted volatiles from nectar. Notably, recent research has shown that SPME at a 60 °C extraction temperature yields the most chemically diverse volatiles from alfalfa plants compared to lower temperatures and shorter extraction times (Yang et al. 2021). Therefore, while our findings provide valuable insights, they should be interpreted with caution in an ecological context.

## Conclusion

In summary, our results have shown that both nectar composition and yeast species have a strong impact on nectar volatile profiles, which in turn influence insect behavior. This highlights the essential role of nectar sugar and amino acid content in mediating the foraging behavior of flower-visiting insects. A better understanding of these interactions could provide valuable insights into the nectar foraging habits of parasitoids, and ultimately aid biological pest control.

## Supplementary Information

Below is the link to the electronic supplementary material.

Supplementary file1 (DOCX 318 KB)

## Data Availability

Data is provided within the manuscript or supplementary information files.
