# Supplementary material for: Impact of Nectar Composition and Nectar Yeasts on Volatile Emissions and Parasitoid Behavior
Source: J Chem Ecol. 2025 Mar 6;51(2):29. doi: 10.1007/s10886-025-01587-1 (PMC11885403; doi:10.1007/s10886-025-01587-1)
Supplement: Supplementary file 1 — Supplementary file1 (DOCX 318 KB) [file 10886_2025_1587_MOESM1_ESM.docx]

IMPACT OF NECTAR COMPOSITION AND NECTAR YEASTS ON VOLATILE EMISSIONS AND PARASITOID BEHAVIOR

ISLAM S. SOBHY^1,2,#,*^, TIM GOELEN^1, $^, FELIX WÄCKERS^3,4^, KEVIN J. VERSTREPEN^5,6^, TOM WENSELEERS^7^, HANS JACQUEMYN^8,9^, AND BART LIEVENS^1,9,*^

*^1^CMPG Laboratory for Process Microbial Ecology and Bioinspirational Management (PME&BIM), Department of Microbial and Molecular Systems, KU Leuven, Leuven, Belgium*

*^2^Department of Plant Protection, Faculty of Agriculture, Suez Canal University, Ismailia, Egypt*

*^3^Biobest, Westerlo, Belgium*

*^4^Lancaster Environment Centre, Lancaster University, Lancaster, United Kingdom*

*^5^VIB – KU Leuven Center for Microbiology, Leuven, Belgium*

*^6^CMPG Laboratory of Genetics and Genomics, Department of Microbial and Molecular Systems, KU Leuven, Leuven, Belgium*

*^7^Laboratory of Socio-Ecology & Social Evolution, Biology Department, KU Leuven, Leuven, Belgium*

*^8^Laboratory of Plant Conservation and Population Biology, Biology Department, KU Leuven, Leuven, Belgium*

*^9^KU Leuven Plant Institute (LPI), KU Leuven, Leuven, Belgium*

***# Current address:*** School of Biosciences, Cardiff University, Museum Avenue, Cardiff, United Kingdom

***$ Current address:*** Flemish Institute for Technological Research (VITO), Mol, Belgium

Islam S. Sobhy (ORCiD ID: 0000-0003-4984-1823)

Tim Goelen (ORCiD ID: 0000-0002-0034-6197)

Felix Wäckers (ORCiD ID: 0000-0002-9748-0615)

Kevin Verstrepen (ORCiD ID: 0000-0002-3077-6219)

Tom Wenseleers (ORCiD ID: 0000-0002-1434-861X)

Hans Jacquemyn (ORCiD ID: 0000-0001-9600-5794)

Bart Lievens (ORCiD ID: 0000-0002-7698-6641)

*** Correspondence:**

Islam S. Sobhy (Email: [sobhyi@cardiff.ac.uk](mailto:sobhyi@cardiff.ac.uk); [is_sobhy@yahoo.com](mailto:is_sobhy@yahoo.com))

Bart Lievens (Email: [bart.lievens@kuleuven.be](mailto:bart.lievens@kuleuven.be))

**Table S1.** Composition of the four synthetic nectars used in this study^a^

| **Code** | **Composition** | **Content of 1 liter nectar** |
| --- | --- | --- |
| **LL** | Low amino acid, Low sugar | 63.2 µL of stock amino acid solution (1g /10 mL) (AMRESCO® code 5851)  150 g of sucrose 99%  1 L H_2_O |
| **LH** | Low amino acid, High sugar | 63.2 µL of stock amino acid solution (1g /10 mL)  500 g of sucrose 99%  1 L H_2_O |
| **HL** | High amino acid, Low sugar | 63.2 mL of stock amino acid solution (1g /10 mL)  150 g of sucrose 99%  1 L H_2_O |
| **HH** | High amino acid, High sugar | 63.2 mL of stock amino acid solution (1g /10 mL)  500 g of sucrose 99%  1 L H_2_O |

^a^All nectar solutions were filtered through a 0.2 µm filter prior to usage.

**Table S2.** Volatile organic compound (VOC) composition^*^ of the cell-free nectars fermented by the nectar yeasts used in this study.

| **Volatiles** | **RI** | **HH** | | |  | **HL** | | |  | **LH** | | |  | **LL** | | |
| --- | --- | --- | --- | --- | --- | --- | --- | --- | --- | --- | --- | --- | --- | --- | --- | --- |
|  |  | **Contr.** | **Mg** | **Mr** |  | **Control** | **Mg** | **Mr** |  | **Contr.** | **Mg** | **Mr** |  | **Contr.** | **Mg** | **Mr** |
| **Alcohols** |  |  |  |  |  |  |  |  |  |  |  |  |  |  |  |  |
| ethanol | 445 | ND | 140±47.8 | 162±6.3 |  | ND | 257±19.3 | 253±8.3 |  | ND | 206±2.2 | 227±12.4 |  | ND | 255±10.9 | 270±10.7 |
| isopropyl alcohol | 536 | ND | 575.6±33.1 | 516.5±8.3 |  | ND | 1097±79.8 | 1108±33.3 |  | ND | 944±54.4 | 946.0±30.4 |  | ND | 924±22.3 | 1088.2±23.1 |
| isobutanol | 653 | ND | 70.1±20.8 | 18.7±7.7 |  | ND | 60.1±7.5 | 52.9±36 |  | ND | 120±6.0 | 27.3±22.3 |  | ND | 9.9±8.0 | 109±9.5 |
| 2,3-butanediol | 753 | ND | 57.3±14.8 | 44.5±2.2 |  | ND | 81.9±25.6 | 110±3.8 |  | ND | 9.6±7.8 | ND |  | ND | 79.6±5.9 | 103±6.5 |
| 3-methyl-2-hexanol | 832 | ND | 46.2±3.1 | 41.9±2.2 |  | ND | 32.3±7.3 | 42.7±3.8 |  | ND | 47.2±13.9 | 50±4.8 |  | ND | 46.9±1.3 | 53±4.1 |
| 4-methyl-1-pentanol | 868 | ND | 6732±1531 | 8966±1567 |  | ND | 9488±485 | 11355±3662 |  | 30.5±4.9 | 7261±155 | 7211±515 |  | ND | 14681±4600 | 12916±2578 |
| 5-methyl-2-furanmethanol | 975 | ND | 3019±557 | 2070±337 |  | ND | 2186±802 | 3131±163 |  | ND | 1917±185 | ND |  | ND | 4018±636 | 2248±1142 |
| 1-octen-3-ol | 981 | ND | 248±22.6 | 243±278 |  | ND | 340±17.8 | 357±10.3 |  | ND | 272±17.8 | 5056±390 |  | ND | 505.4±13.3 | 9255±7157 |
| 2-ethyl-1-hexanol | 1031 | ND | 12616±1643 | 4329±2105 |  | ND | 12268±752 | 16056±154 |  | ND | ND | 7688±736 |  | ND | 7189±8.30 | ND |
| benzyl alcohol | 1036 | ND | 62.8±26.2 | 96.5±9.0 |  | ND | 1648±1237 | 169±1.8 |  | ND | 676±470 | 108±6.8 |  | ND | 2086±1636 | 199±0.4 |
| phenylethyl alcohol | 1114 | ND | 138±33.7 | 118±13.5 |  | ND | 241±38.5 | 1975±446 |  | ND | 23.4±9.5 | 51.2±9.8 |  | ND | 745±14.4 | 789±3.8 |
| Isoborneol | 1162 | ND | 2284±116.7 | 17037±9095 |  | ND | 4347±1731 | 36355±4883 |  | ND | 5406±473 | ND |  | ND | ND | 7555±1702 |
| **Benzenoids** |  |  |  |  |  |  |  |  |  |  |  |  |  |  |  |  |
| styrene | 891 | ND | 188±12.4 | 173±12.8 |  | ND | 136±13.5 | 128±5.1 |  | ND | 567±148 | 613±35.2 |  | ND | 118±4.3 | 138±4.1 |
| *E*-methyl isoeugenol | 1499 | ND | 2271±44.2 | 2646±320 |  | ND | 3084±144 | 3788±14.6 |  | 288±54.4 | 2922±56.7 | 1369±377 |  | ND | 3395±78.2 | 3572±140 |
| **Esters** |  |  |  |  |  |  |  |  |  |  |  |  |  |  |  |  |
| ethyl acetate | 613 | ND | 100±4.2 | 115±8.8 |  | ND | 172±5.7 | 167±16.3 |  | ND | 60.4±2.0 | 34.6±11 |  | ND | 130±54.6 | 217±12.5 |
| propyl acetate | 712 | ND | 194±2.4 | 193±17.6 |  | ND | 78±1.5 | 78.8±4.4 |  | ND | 159±60.3 | 875±60.6 |  | ND | 64.8±6.4 | 66.3±1.5 |
| ethyl isobutyrate | 755 | ND | 69.2±5.2 | 281±19.9 |  | ND | 118±34.6 | 194±6.9 |  | ND | 627±194 | 241±14.6 |  | ND | 139±57.1 | 129±31.7 |
| isobutyl acetate | 780 | ND | ND | 297±48.3 |  | ND | 212±4.4 | 71.3±21.9 |  | ND | 46.7±5.2 | 310±18.6 |  | ND | 46.9±5.8 | ND |
| amyl acetate | 916 | ND | 30.5±1.9 | 27.4±6.1 |  | ND | 59±5.3 | 61.4±4.4 |  | ND | 33.3±2.5 | 19.8±8.1 |  | ND^c^ | 65.4±15.7 | 64.1±6.6 |
| ethyl tiglate | 926 | ND | 151±13.1 | 247±156 |  | ND | 317±53.5 | 293±47.1 |  | ND | 40.8±16.8 | 68.6±1.8 |  | 20.6±9.0 | 160±14.2 | 215±10.8 |
| isopentyl butanoate | 1041 | ND | 13582±2890 | 7831±858 |  | ND | 1238±654 | 9375±767 |  | ND | 7353±610 | 6258±5093 |  | ND | 10473±4070 | 9979±411 |
| prenyl isobutyrate | 1053 | ND | 43.5±8.5 | 212±21.2 |  | ND | 344±216 | 86.5±16.1 |  | ND | 40.5±33.1 | ND |  | ND | ND | 27.1±11.9 |
| ethyl-(*E*)-cinnamate | 1443 | ND | 206±9.3 | 127±4.4 |  | ND | 310±22.1 | 271±23.0 |  | ND | 71.8±11.9 | ND |  | ND | ND | ND |
| pentyl octanoate | 1468 | ND | 182±10.5 | 188±20.4 |  | ND | 179±12.7 | 195±6.7 |  | 98.8 ± 7.1 | 209±18.0 | 501±36.6 |  | ND | 118±22.9 | 166±12.9 |
| ethyl dodecanoate | 1582 | ND | 796±68.9 | 934±147 |  | ND | 676±42.7 | 760±25.9 |  | ND | 1052±192 | 932±289 |  | ND | ND | 708±20.0 |
| isopropyl-hexadecanoate | 1827 | ND | 86.6±12.3 | 110±38.0 |  | ND | ND | 140±17.7 |  | ND | ND^c^ | 62.7±10.5 |  | ND | 194±95.4 | 152±35.1 |
| **Terpenoids** |  |  |  |  |  |  |  |  |  |  |  |  |  |  |  |  |
| (*E*)-β-ocimene | 1051 | ND | 63.5±6.2 | ND |  | ND | 861±615 | 125±35.6 |  | ND | 98.7±41.2 | 211±66.2 |  | ND | 549±367 | 126±11.0 |
| α-terpineol | 1143 | ND | 270±24.4 | 273±22.7 |  | ND | 561±216 | 582±27.4 |  | ND | 414±44.3 | 378±9.7 |  | ND | 725±19.5 | 538±195 |
| α-guaiene | 1436 | ND | 84.4±17.4 | 132±31.5 |  | ND | 70.9±3.2 | 86.4±5.3 |  | ND | 159±37.9 | 256±17.0 |  | ND | 73.1±8.4 | 97.8±2.4 |
| trans-calamenene | 1508 | ND | 3223±32.1 | 3020±1067 |  | ND | ND | 5401±39.1 |  | ND | 282±63.7 | 137±35.1 |  | ND | 1184±211 | 2692±1021 |
| 9-epi-(*E*)-caryophyllene | 1677 | ND | 273±56.1 | 362±48.5 |  | ND | 590±54.1 | 710±60.2 |  | ND | 381±10.5 | 31.7±12.9 |  | 98.7±3.8 | 1090±257 | 1273±56.1 |
| 14-hydroxy-alpha-humulene | 1724 | ND | 339±106 | ND |  | ND | 363±40.6 | 469±40.8 |  | ND | 360±46.6 | ND |  | ND | 290±120 | 529±92.6 |
| **Miscellaneous** |  |  |  |  |  |  |  |  |  |  |  |  |  |  |  |  |
| 2-butanone | 597 | ND | 649±53.1 | 1042±108 |  | ND | 2387±53.8 | 2781±91.4 |  | ND | 534±42.2 | 646±11.6 |  | ND | 1972±814 | ND |
| methylpyrazine | 831 | ND | 73.0±3.9 | 45.1±18.4 |  | ND | 97.9±9.6 | 86.8±5.3 |  | ND | ND | ND |  | ND | 62.2±0.4 | 58.6±1.4 |
| benzaldehyde | 953 | ND | 277±112 | 663±295 |  | ND | 267±89.3 | 289±33.9 |  | ND | 98.8±7.2 | 123±14.5 |  | ND | 246±34.1 | 172±70.6 |
| undecane | 1100 | 25.3±20.7 | 54.7±7.1 | 37.1±15.9 |  | 50.6±20.7 | 79.5±7.1 | 74.3±0.3 |  | ND | ND | ND |  | 39.9±17 | 97.4±8.6 | 116±3.9 |
| **Total** | | 25.3±20.7 | 49264±2801 | 52715±13174 |  | 50.6±20.7 | 44335±2868 | 97249±7685 |  | 417.4±50.6 | 32578±1144 | 34503±4699 |  | 159.2±28.4 | 51436±6982 | 55712±8978 |

*Peak areas and Kovats retention indices (RI) were obtained using a MXT-5 equipped GC-MS. Presented values are means of peak areas (x 10^7^) ± SE of three biological replicates (*n* = 3) of different nectars (HH: high amino acid and high sugar content; HL: high amino acid and low sugar content; LH: low amino acid and high sugar content; LL: low amino acid and low sugar content). These nectars were either fermented by two specialist nectar yeasts—*Metschnikowia gruessii* (Mg) and *Metschnikowia reukaufii* (Mr) - or left unfermented (Control)*.* Under each chemical class, VOCs are ordered in accordance with their increasing retention time in the gas chromatograph and retention index. VOCs were tentatively identified using their spectra, Kovats retention indices and matches from the NIST2017, FFNSC, and Adams libraries.

**
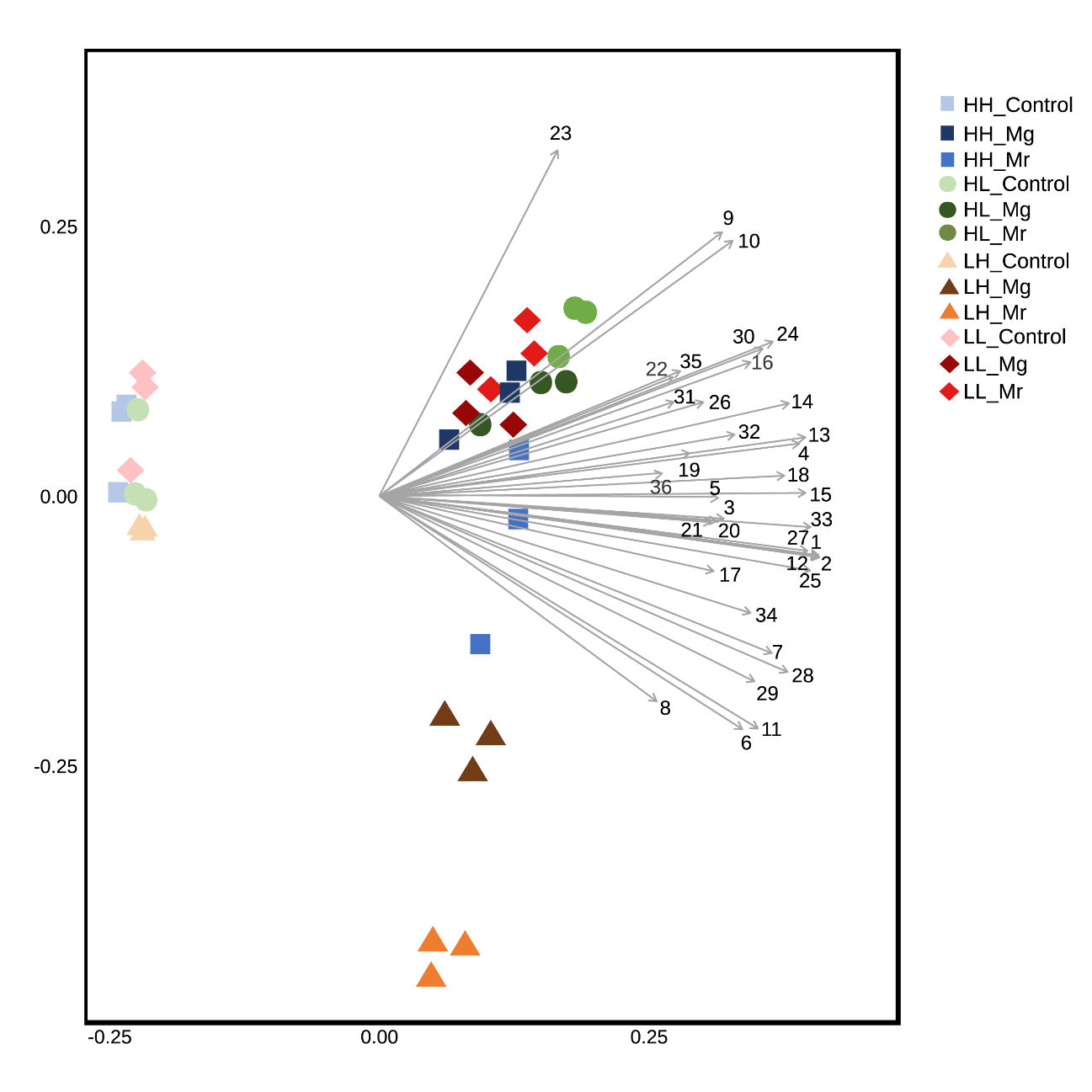
**

**PC1 (65.77 %)**

**PC2 (8.77 %)**

**Figure S1.** Principal Component Analysis (PCA) of the volatile profiles emitted from different synthetic nectars (HH, high amino acid and high sugar content; HL, high amino acid and low sugar content; LH, low amino acid and high sugar content; LL, low amino acid and low sugar content), which were fermented by either *Metschnikowia gruessii* (Mg) or *M. reukaufii* (Mr) or not fermented (Control). The Biplot visualizes the ordination of collected samples according to the first two PCs based on the quantity of the different volatiles emitted from the different nectars, whereas vectors (in grey) visualize the loadings for each variable. Vector numbers refer to the different volatile compounds: (1) isopropylalcohol, (2) ethanol, (3) 2-butanone, (4) ethyl acetate, (5) isobutanol, (6) propyl acetate, (7) ethyl isobutyrate, (8) isobutyl acetate, (9) methylpyrazine, (10) 2,3-butanediol, (11) styrene, (12) 3-methyl-2-hexanol, (13) amyl acetate, (14) ethyl tiglate, (15) benzaldehyde, (16) 5-methyl-2-furanmethanol, (17) 1-octen-3-ol, (18) isopentyl butanoate, (19) 2-ethyl-1-hexanol, (20) benzyl alcohol, (21) (*E*)-beta-ocimene, (22) prenyl isobutyrate, (23) undecane, (24) phenethyl alcohol, (25) 4-methyl-1-pentanol, (26) isoborneol, (27) 1-alpha-terpineol, (28) α-guaiene, (29) pentyl-octanoate, (30) 9-epi-(*E*)-caryophyllene, (31) ethyl-(*E*)-cinnamate, (32) trans-calamenene, (33) E-methyl isoeugenol, (34) ethyl dodecanoate, (35) 14-hydroxy-alpha-humulene, (36) isopropyl-hexadecanoate. All analyses were performed on cell-free nectar solutions (three biological replicates; *n* = 3). Volatile data were log-transformed and auto-scaled (mean-centered and divided by the standard deviation of each variable) prior to analysis.

**
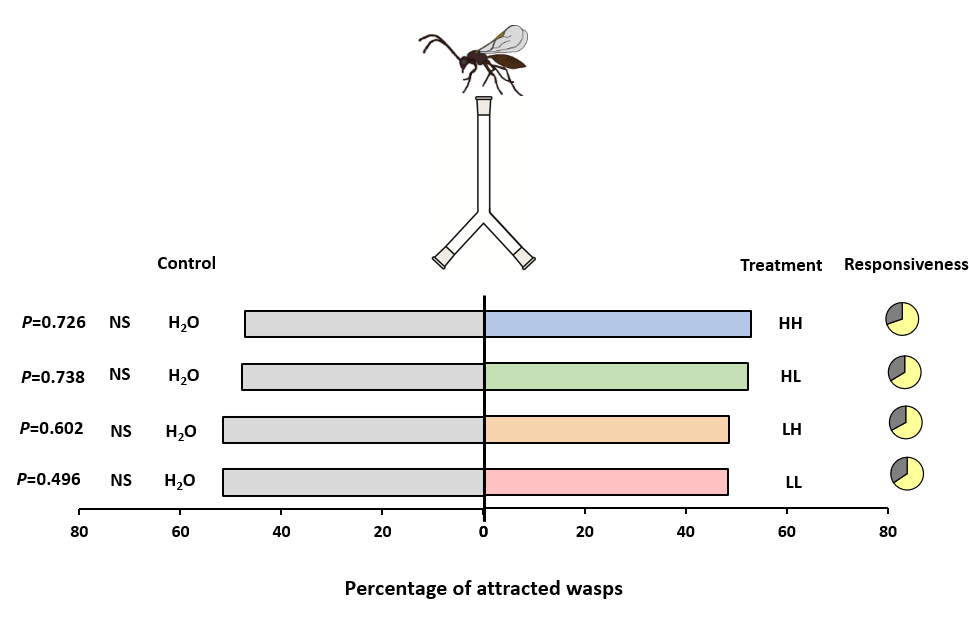
**

**Figure S2.** Olfactory response of *Aphidius ervi* females when given the choice between water and non-inoculated, non-fermented nectar (percentage ± SE, *n* = 40). Treatments included four artificial nectars (HH, high amino acid and high sugar content; HL, high amino acid and low sugar content; LH, low amino acid and high sugar content; and LL, low amino acid and low sugar content). The bioassay was carried out by releasing 40 groups of five naïve (inexperienced to smell and food) females at the base of a two-choice Y-tube olfactometer and evaluating their response ten minutes after their release. Pie charts show the distribution of responding (in yellow) and non-responding (in grey) individuals. Non-responders were eliminated from statistical analysis. No statistical differences were seen between any of the water and nectar treatments (Generalized Linear Mixed Model) (NS, non-significant).
